# Interpreting EORTC CAT Core short-form scores in metastatic breast cancer: EQ-VAS-associated cut-points and comparison with published thresholds for clinical importance

**DOI:** 10.1186/s41687-026-01254-3

**Published:** 2026-11-06

**Authors:** Pimrapat Gebert, Maria Margarete Karsten, Ulrike Grittner, Morten Aagaard Petersen, Lea Doppelbauer, Felix Fischer, Matthias Rose, Christoph Kowalski, Therese Pross

**Affiliations:** 1 Institute of Biometry and Clinical Epidemiology, Charité - Universitätsmedizin Berlin, Berlin, Germany; 2 Berlin Institute of Health at Charité - Universitätsmedizin Berlin, Berlin, Germany; 3 Department of Gynecology with Breast Center, Charité - Universitätsmedizin Berlin, Berlin, Germany; 4 Department of Geriatrics and Palliative Medicine GP, Bispebjerg and Frederiksberg Hospital, University of Copenhagen, Copenhagen, Denmark; 5 Department for Psychosomatic Medicine, Charité - Universitätsmedizin Berlin, Berlin, Germany; 6 German Cancer Society, Berlin, Germany; 7 BIH Biomedical Innovation Academy, BIH Charité Digital Clinician Scientist Program, Berlin Institute of Health at Charité - Universitätsmedizin Berlin, Berlin, Germany

**Keywords:** Metastatic breast cancer, EORTC CAT Core, Short forms, Patient-reported outcomes, EQ-VAS, Cut-points

## Abstract

**Background:**

Published thresholds for clinical importance (TCIs) for the European Organisation for Research and Treatment of Cancer CAT Core were derived using domain-specific relevance anchors, which are not routinely collected. We estimated EQ-VAS-associated cut-points for EORTC CAT Core short-form scores in patients with metastatic breast cancer, compared them with published TCIs, and examined patient-reported Eastern Cooperative Oncology Group (ECOG) performance status as a secondary reference classification.

**Methods:**

This post hoc cross-sectional analysis used data from the PRO B trial. EQ-VAS ≤ 70 was used to define lower global health status, with EQ-VAS ≤ 65 examined in sensitivity analysis; patient-reported ECOG ≥ 2 defined impaired performance status in secondary analysis. Receiver operating characteristic (ROC) analyses based on continuous CAT Core T-scores identified Youden-optimal cut-points with 5,000 bootstrap replications for estimating confidence intervals and optimism-corrected performance.

**Results:**

Among 855 patients with CAT Core data available for the baseline analysis (mean age 51 years, SD 11), 841 had EQ-VAS and 854 had ECOG data. Using EQ-VAS ≤ 70, AUCs ranged from 0.57 to 0.82; eight of 14 domains had AUC ≥ 0.70, five had AUC 0.60–0.69, and diarrhea had AUC 0.57. Four domains did not meet the criterion used for interpretation of |ρ| ≥0.30 and were therefore interpreted as exploratory. Published TCIs showed different sensitivity-specificity profiles when evaluating against the EQ-VAS reference classification, illustrating the dependence of cut-point performance on the reference construct.

**Conclusions:**

This study identified EQ-VAS-associated cut-points for EORTC CAT Core short-form scores in metastatic breast cancer. Their performance varied by domain, and they should be interpreted as context-specific reference values rather than replacements for published relevance-based TCIs.

**Supplementary Information:**

The online version contains supplementary material available at https://doi.org/10.1186/s41687-026-01254-3.

## Background

Health-related quality of life (HRQoL) is commonly assessed in oncology using patient-reported outcomes (PROs), in particular to support evaluation of treatment impact and patient-centered care [1–4]. The most frequently used instrument is the EORTC QLQ-C30, developed by the European Organisation for Research and Treatment of Cancer (EORTC) and covering multiple functional and symptom scales. Building on the QLQ-C30 framework, the EORTC computer adaptive testing (CAT) Core was developed to improve the precision and efficiency while maintaining comparability with the EORTC QLQ-C30 [5–7]. CAT Core scores are reported on a standardized T-score metric; however, interpretation of score levels in terms of clinical relevance remains challenging [8].

Thresholds for clinical importance (TCIs) have previously been estimated for the EORTC CAT Core using domain-specific patient-reported relevance anchors designed to capture whether health problems are important to patients [9]. EuroQol visual analogue scale (EQ-VAS) and patient-reported Eastern Cooperative Oncology Group (ECOG) instead measure global health and performance status and do not directly assess domain-specific clinical importance.

We therefore estimated EQ-VAS-associated cut-points for EORTC CAT Core short-form scores in patients with metastatic breast cancer (mBC), examined patient-reported ECOG as a secondary reference classification, and compared the resulting cut-points descriptively with published CAT Core TCIs.

## Methods

The data were derived from the PRO B trial, a multicenter randomized controlled study that enrolled 924 patients with mBC from 52 German centers [10]. The trial assessed HRQoL using fixed forms derived from the EORTC CAT Core item banks [11] and the German version of the EQ-5D-5 L [12]. For the present analysis, the baseline CAT Core assessment was defined as the first available questionnaire within the study’s baseline completion window. Participants in the control group received the long form and could complete the baseline questionnaire within seven days. Participants in the intervention group received the short form at week 0; for 39 intervention-group participants without a week-0 response, the week-1 questionnaire was used, at which time the long form was administered. Consequently, 391 short-form and 464 long-form assessments were included for domains in which the form differed. Fixed forms rather than computer-adaptive item selection were used.

The short and long versions differed for physical functioning (5 vs. 8 items), emotional functioning (4 vs. 8), cognitive functioning (4 vs. 8), social functioning (4 vs. 8), and pain (2 vs. 8); the same baseline forms were administered for role functioning (8 items), fatigue (8 items), and the remaining symptom domains (2 items each) (Table S1). Because all fixed forms are calibrated on the same EORTC CAT Core T-score metric, data were pooled for the primary analyses. To assess the potential influence of the administered form, sensitivity analyses were conducted separately for the short and long forms for the five domains in which the forms differed.

The CAT Core domains were scored using the corresponding EORTC CAT Core item-response theory scoring tables and expressed as standardized T-scores, with 50 being the mean and 10 the standard deviation in the general European population [6]. Higher scores on functioning domains indicate better functioning, whereas higher scores on symptom domains indicate greater symptom burden. Before analysis, all 14 functioning and symptom domain scores were checked against the corresponding EORTC CAT Core IRT scoring tables, including item coding, scoring direction, and calculated T-scores. Global health status/QoL (GH/QoL) was calculated from the two original QLQ-C30 global health/quality-of-life items using the EORTC-provided T-score scoring table and was summarized descriptively but excluded from cut-point derivation because the published CAT Core TCIs concern the 14 functioning and symptom domains and because GH/QoL overlaps conceptually with the EQ-VAS.

### Reference classifications for cut-point derivation

Baseline EQ-VAS, ranging from 0 (worst imaginable health) to 100 (best imaginable health) [12], was used as the primary reference measure. Because no validated EQ-VAS threshold for domain-specific clinical importance in mBC was identified, EQ-VAS was categorized as lower global health status (≤ 70) and higher global health status (> 70), informed by EQ-VAS distributions reported in breast cancer populations [13, 14]. This categorization was treated as a global-health reference classification rather than a domain-specific clinical importance anchor. A sensitivity analysis used EQ-VAS ≤ 65, corresponding approximately to the cohort mean.

Patient-reported ECOG performance status [15] was examined as a secondary reference classification and dichotomized as impaired (ECOG ≥ 2) versus good performance status (ECOG 0–1). Patient-reported ECOG was considered secondary because it primarily reflects functional performance rather than domain-specific relevance. Additionally, we compared the resulting cut-points with those derived from the global health anchor and the current EORTC CAT TCIs based on Giesinger et al. [9].

### Statistical analysis

This was a post hoc secondary cross-sectional analysis of baseline PRO B trial data. Descriptive statistics were used to summarize patient characteristics and demographic data. Participants without baseline CAT Core data were excluded from the analysis cohort, and reference-specific analyses used complete cases for the corresponding reference measure.

Associations between CAT Core T-scores and the original continuous EQ-VAS and patient-reported ECOG scores were assessed using Spearman rank correlations with bootstrap 95% confidence intervals (CIs). Because no anchor-strength criterion was prespecified, an absolute Spearman correlation of |ρ| ≥0.30 was applied post hoc as a pragmatic criterion for interpretation [16]; cut-points for domains below this criterion were retained for transparency but interpreted as exploratory.

Group separation was summarized using Cohen’s d, with values of approximately 0.2, 0.5, and 0.8 conventionally representing small, moderate, and large standardized differences, respectively [17]. Receiver operating characteristic (ROC) curve analyses were conducted using the continuous CAT Core T-scores, and cut-points were selected by maximizing the Youden index [18] with 5,000 bootstrap replications to obtain 95% percentile bootstrap CIs for cut-points and areas under the curve (AUCs). Sensitivity, specificity, positive predictive value (PPV), and negative predictive value (NPV) at the derived cut-points were internally validated using bootstrap optimism correction. According to commonly used criteria, AUC values between 0.70 and 0.80 indicate acceptable discrimination, whereas values between 0.60 and 0.70 indicate moderate discrimination [19]. Published CAT Core TCIs were applied without modification to the PRO B cohort and evaluated against the EQ-VAS ≤ 70 reference classification.

We conducted all analyses and visualizations using Stata MP/19 (StataCorp, 2025, College Station, Texas, USA).

## Results

Of 924 randomized participants, 855 (92.5%) had CAT Core data available for the baseline analysis; 69 had no CAT Core data available within the baseline completion window. Baseline characteristics of participants with and without baseline CAT Core data were broadly similar (Table S2). Among the 855 participants, 841 had complete EQ-VAS data and 854 had complete patient-reported ECOG data. Baseline characteristics are shown in Table 1.

**Table 1:** Baseline demographic, clinical, and patient-reported characteristics

Baseline parameters	Overall (*n* = 855)	
Age at randomization (years) – Mean (SD)	51.0 (10.9)	
Female sex, n (%)	855 (100.0%)	
Family status		
Single	109 (12.9%)	
Married	555 (65.5%)	
Separate/Divorced/Widowed	183 (21.6%)	
Missing, number	8	
Having children < 14 years	136 (16.1%)	
Education		
Low	79 (9.3%)	
Middle	464 (54.8%)	
High	304 (35.9%)	
Missing, number	8	
Employed status	452 (53.4%)	
History of depression	86 (10.2%)	
NCI comorbidity index – mean (SD)	0.1 (0.3)	
Time since metastatic diagnosis (months) – median (IQR)	17 (4–43)	
Metastatic presentation^1^		
De novo	349 (40.8%)	
Recurrent	502 (58.7%)	
Unknown	4 (0.5%)	
Clinical subtype		
HR+/HER2+	130 (16.5%)	
HR+/HER2-	528 (66.8%)	
HR-/HER2+	51 (6.5%)	
TNBC	81 (10.3%)	
Missing, number	65	
Site of metastases		
Brain or multiple metastases^ 2^	477 (55.8%)	
Bone, lymph nodes, or skin	252 (29.5%)	
Visceral (only one organ)	126 (14.7%)	
Systemic therapy at baseline		
Endocrine	505 (59.1%)	
Chemotherapy	410 (48.0%)	
Anti-HER2	194 (22.7%)	
Another immunotherapy	28 (3.3%)	
Targeted therapy	302 (35.3%)	
Other	116 (13.6%)	
**Patient-reported outcomes and health status at baseline**	**Mean (SD)**	**Median (IQR)**
**EORTC CAT Core scales**		
Global health status/QoL	47.2 (9.0)	49.4 (42.9–53.0)
Functioning scales		
Physical functioning	41.2 (9.4)	41.2 (34.7–46.4)
Role functioning	40.3 (10.2)	39.6 (33.7–45.9)
Emotional functioning	41.3 (8.8)	40.3 (35.4–47.1)
Cognitive functioning	43.7 (10.0)	42.7 (37.2–51.6)
Social functioning	41.3 (8.9)	41.0 (35.0-47.3)
Symptom scales		
Fatigue	58.8 (9.4)	58.5 (53.3–64.7)
Nausea/vomiting	59.4 (10.9)	51.7 (51.7–64.4)
Pain	54.8 (10.0)	55.6 (44.9–63.6)
Dyspnea	58.4 (10.6)	59.3 (53.8–67.2)
Sleep disturbances	57.4 (8.6)	55.3 (51.9–63.0)
Appetite loss	55.0 (11.6)	45.9 (45.9–64.6)
Constipation	53.0 (10.9)	43.6 (43.6–59.7)
Diarrhea	55.3 (11.7)	45.2 (45.2–64.2)
Financial impact	54.0 (10.5)	46.1 (46.1–63.1)
**EQ-VAS (0–100)**		
Mean (SD)	65.0 (20.5)	
Median (IQR)	69 (49–80)	
Missing, number	14	
**Patient-reported ECOG performance status**	**Number (%)**	
ECOG 0	181 (21.2%)	
ECOG 1	468 (54.8%)	
ECOG 2	162 (19.0%)	
ECOG 3	41 (4.8%)	
ECOG 4	2 (0.2%)	
Missing, number	1	

Percentages are based on participants with available data for the respective variable; missing values are reported separately where applicable

^1^Classification based on timing of first documented distant metastasis relative to initial diagnosis (≤ 90 days = de novo; >90 days = recurrent)

^2^ Site of metastases categories correspond to the metastatic-site stratification variable used in the PRO B trial

ECOG, Eastern Cooperative Oncology Group; EORTC, European Organisation for Research and Treatment of Cancer; EQ-VAS, EuroQol visual analogue scale; HER2, human epidermal growth factor receptor 2; HR, hormone receptor; IQR, interquartile range; NCI, National Cancer Institute; QoL, quality of life; SD, standard deviation; TNBC, triple-negative breast cancer

**Fig. 1:**
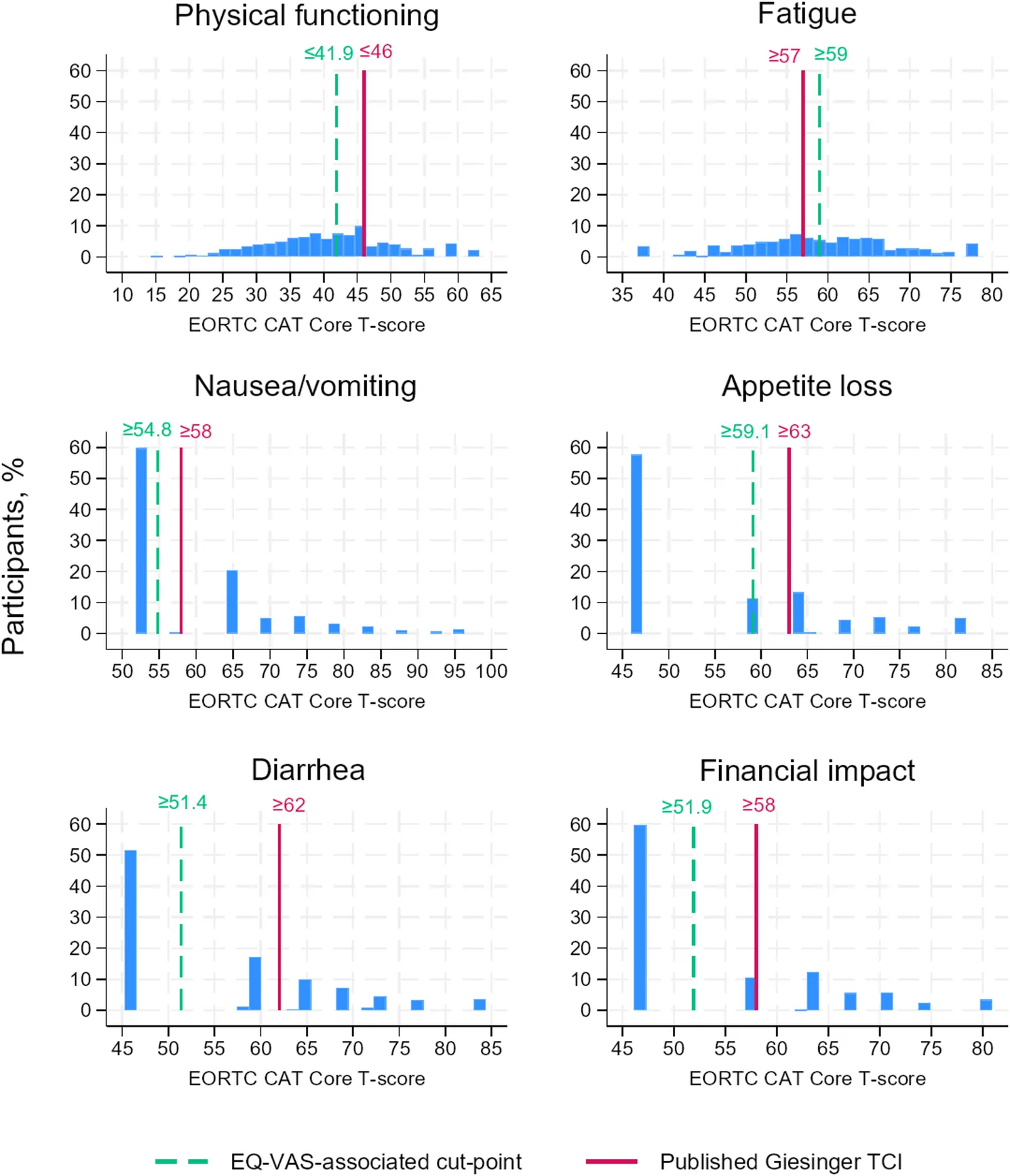
Distribution of selected EORTC CAT Core T-scores used in the baseline analysis with EQ-VAS-associated cut-points and published thresholds for clinical importance. Histograms show the percentage distribution of the EORTC CAT Core T-scores. Green dashed lines indicate EQ-VAS-associated cut-points derived using EQ-VAS ≤ 70 as the primary reference classification; solid magenta lines indicate published thresholds for clinical importance from Giesinger et al. Threshold values and classification directions are shown above each line. Physical functioning and fatigue illustrate domains with broadly distributed scores, whereas nausea/vomiting, appetite loss, diarrhea, and financial impact illustrate domains with substantial concentration at the observed minimum. Lower physical functioning scores indicate worse functioning, whereas higher symptom scores indicate greater symptom burden. Published thresholds were originally derived using domain-specific relevance anchors and are shown here for descriptive comparison

Score distributions were highly concentrated for several symptom domains. At least 50% of participants had the observed minimum score for nausea/vomiting (59.8%), appetite loss (57.7%), constipation (50.4%), diarrhea (51.7%), and financial impact (59.6%) (Table S3; Fig. 1).

All five functioning domains and five of nine symptom domains met the criterion used for interpretation of |ρ| ≥0.30. Standardized mean differences (Cohen’s d) for the EQ-VAS-defined global health status groups ranged between 0.28 and 1.25 and patient-reported ECOG performance status ranged between 0.21 and 1.14 (Table 2).

**Table 2:** EORTC CAT Core scores by EQ-VAS and patient-reported ECOG reference classifications and correlations with the continuous measures

EORTC CAT Core scale	Global health status – Mean (SD)				Patient-reported ECOG performance status – Mean (SD)			
	Lower (EQ-VAS ≤ 70) (*n* = 444)	High (EQ-VAS > 70) (*n* = 397)	Cohen’s d effect size	Spearman ρ (95% bootstrap CI) ^1^	Impaired (ECOG ≥ 2) (*n* = 205)	Good (ECOG 0–1) (*n* = 649)	Cohen’s d effect size	Spearman ρ (95% bootstrap CI)^1^
Functioning domain								
Physical functioning	36.7 (7.8)	46.6 (8.1)	1.25	0.601 (0.552, 0.646)	33.9 (7.3)	43.5 (8.8)	1.14	-0.612 (-0.656, -0.563)
Role functioning	35.4 (8.7)	46.0 (8.6)	1.23	0.619 (0.573, 0.661)	32.6 (8.6)	42.7 (9.4)	1.10	-0.595 (-0.640, -0.543)
Emotional functioning	38.0 (7.7)	45.3 (8.2)	0.91	0.491 (0.435, 0.543)	36.5 (7.2)	42.9 (8.7)	0.77	-0.366 (-0.420, -0.307)
Cognitive functioning	40.4 (9.6)	47.5 (9.1)	0.76	0.417 (0.357, 0.470)	38.8 (9.8)	45.2 (9.6)	0.67	-0.351 (-0.409, -0.290)
Social functioning	37.6 (7.7)	45.7 (8.2)	1.03	0.526 (0.472, 0.575)	35.9 (7.3)	43.1 (8.7)	0.86	-0.468 (-0.521, -0.410)
Symptom domain								
Fatigue	63.2 (8.1)	53.8 (8.1)	1.16	-0.601 (-0.643, -0.556)	65.2 (7.5)	56.8 (9.0)	0.96	0.505 (0.451, 0.553)
Nausea/vomiting	62.3 (11.8)	56.1 (8.6)	0.59	-0.334 (-0.392, -0.274)	64.7 (13.2)	57.7 (9.5)	0.67	0.308 (0.243, 0.369)
Pain	58.3 (9.8)	50.7 (8.5)	0.82	-0.465 (-0.519, -0.409)	60.2 (9.7)	53.1 (9.4)	0.76	0.420 (0.361, 0.478)
Dyspnea	62.1 (9.8)	54.0 (9.9)	0.82	-0.454 (-0.508, -0.398)	64.3 (9.2)	56.4 (10.3)	0.79	0.453 (0.398, 0.506)
Sleep disturbances	59.1 (8.6)	55.5 (8.3)	0.42	-0.267 (-0.330, -0.202)	60.0 (8.4)	56.6 (8.5)	0.40	0.235 (0.170, 0.297)
Appetite loss	57.7 (12.4)	51.6 (9.5)	0.54	-0.323 (-0.382, -0.263)	60.8 (12.9)	53.1 (10.6)	0.69	0.312 (0.251, 0.371)
Constipation	54.9 (11.8)	50.7 (9.3)	0.40	-0.210 (-0.272, -0.144)	57.0 (11.8)	51.7 (10.3)	0.50	0.226 (0.159, 0.290)
Diarrhea	56.9 (12.1)	53.6 (11.0)	0.28	-0.162 (-0.227, -0.095)	57.2 (12.0)	54.7 (11.6)	0.21	0.142 (0.077, 0.205)
Financial impact	56.4 (11.5)	51.3 (8.7)	0.50	-0.266 (-0.329, -0.199)	58.6 (12.3)	52.5 (9.4)	0.60	0.270 (0.204, 0.334)

CAT, computer adaptive testing; ECOG, Eastern Cooperative Oncology Group; EQ-VAS, EuroQol visual analogue scale; EORTC, European Organisation for Research and Treatment of Cancer; SD, standard deviation. ¹Spearman rank correlations were performed using the original continuous EQ-VAS and ECOG scales. Higher CAT Core scores indicate better functioning for functioning domains and greater symptom burden for symptom domains; higher EQ-VAS scores indicate better global health, whereas higher ECOG scores indicate worse performance status. Cohen’s *d* values are presented as absolute standardized mean differences

Using EQ-VAS ≤ 70 as the primary reference classification, AUCs based on continuous CAT Core T-scores ranged from 0.57 to 0.82. Eight of 14 domains had AUCs ≥ 0.70, five had AUCs between 0.60 and 0.69, and diarrhea showed low discrimination (AUC 0.57). EQ-VAS-associated cut-points ranged from ≤ 38.1 to ≤ 41.9 for functioning domains and from ≥ 51.4 to ≥ 61.5 for symptom domains (Table 3 and Table S4). The secondary ECOG analyses yielded AUCs ranging from 0.56 to 0.80, with performance varying similarly by domain (Table S5). Sensitivity analyses using EQ-VAS ≤ 65 produced broadly similar patterns (Table S6).

**Table 3:** EQ-VAS-associated cut-points for EORTC CAT Core domains and their performance using EQ-VAS ≤ 70 as the primary reference classification

Domain	Published TCI (Giesinger et al.)	EQ-VAS-associated cut-point (95% bootstrap CI)	Spearman ρ (95% bootstrap CI)	AUC (95% bootstrap CI)	Optimism-corrected performance, %			
					Sensitivity	Specificity	PPV	NPV
**Functioning domain**								
Physical functioning	≤ 46	≤ 41.9 (40.8, 44.4)	0.601 (0.552, 0.646)	0.82 (0.79, 0.84)	77.5	72.6	76.0	74.3
Role functioning	≤ 37	≤ 38.1 (37.5, 41.3)	0.619 (0.573, 0.661)	0.82 (0.79, 0.85)	65.9	83.8	82.1	68.7
Emotional functioning	≤ 46	≤ 39.9 (38.5, 43.5)	0.491 (0.435, 0.543)	0.74 (0.71, 0.78)	64.1	72.7	72.5	64.3
Cognitive functioning	≤ 45	≤ 41.5 (38.2, 46.2)	0.417 (0.357, 0.470)	0.71 (0.67, 0.74)	58.4	71.7	69.8	60.6
Social functioning	≤ 41	≤ 39.5 (37.8, 44.2)	0.526 (0.472, 0.575)	0.77 (0.74, 0.80)	64.4	75.6	74.7	65.5
**Symptom domain**								
Fatigue	≥ 57	≥ 59.0 (56.4, 59.8)	-0.601 (-0.643, -0.556)	0.80 (0.77, 0.83)	71.3	77.2	77.8	70.6
Nausea/vomiting	≥ 58	≥ 54.8 (54.8, 61.1)	-0.334 (-0.392, -0.274)	0.65 (0.62, 0.69)	53.7	75.0	70.6	59.2
Pain	≥ 56	≥ 55.9 (55.5, 58.9)	-0.465 (-0.519, -0.409)	0.72 (0.69, 0.76)	61.6	74.9	73.3	63.6
Dyspnea	≥ 60	≥ 61.5 (53.8, 61.5)	-0.454 (-0.508, -0.398)	0.72 (0.69, 0.76)	54.8	78.2	74.0	60.7
Sleep disturbances	≥ 55	≥ 59.2 (50.9, 65.3)	-0.267 (-0.330, -0.202) ^a^	0.61 (0.58, 0.65)	46.6	67.4	61.5	52.9
Appetite loss	≥ 63	≥ 59.1 (52.3, 62.0)	-0.323 (-0.382, -0.263)	0.63 (0.60, 0.67)	52.1	70.3	66.2	56.8
Constipation	≥ 57	≥ 54.2 (48.4, 66.1)	-0.210 (-0.272, -0.144) ^a^	0.60 (0.56, 0.63)	47.5	66.7	61.3	53.2
Diarrhea	≥ 62	≥ 51.4 (51.4, 64.6)	-0.162 (-0.227, -0.095) ^a^	0.57 (0.54, 0.61)	54.0	58.1	58.9	53.1
Financial impact	≥ 58	≥ 51.9 (51.9, 63.2)	-0.266 (-0.329, -0.199) ^a^	0.62 (0.59, 0.65)	49.8	70.3	65.1	55.7

^a^ Anchor association did not meet the criterion used for interpretation of |ρ| ≥0.30; the corresponding cut-point should be interpreted as exploratory

EQ-VAS ≤ 70 was defined as lower global health status and > 70 as higher global health status. Spearman correlations were calculated using continuous EQ-VAS scores

For functioning domains, lower CAT Core scores indicate worse functioning and cut-points are expressed as ≤; for symptom domains, higher scores indicate greater symptom burden and cut-points are expressed as ≥

Cut-point and AUC confidence intervals are 95% percentile bootstrap confidence intervals based on 5,000 replications. AUCs were calculated using the continuous CAT Core T-scores. Sensitivity, specificity, PPV, and NPV are optimism-corrected point estimates

PPV and NPV are based on the observed prevalence of EQ-VAS ≤ 70 in the study population and may differ in populations with different case mix

Published TCIs were taken from Giesinger et al. and are shown for descriptive comparison only; their original derivation used domain-specific relevance anchors rather than EQ-VAS

AUC, area under the receiver operating characteristic curve; CAT, computer adaptive testing; CI, confidence interval; EQ-VAS, EuroQol visual analogue scale; EORTC, European Organisation for Research and Treatment of Cancer; NPV, negative predictive value; PPV, positive predictive value; TCI, threshold for clinical importance

When published Giesinger TCIs were applied to PRO B and evaluated against EQ-VAS ≤ 70, sensitivity and specificity varied substantially by domain (Table S7). For example, the published physical functioning threshold (≤ 46) demonstrated sensitivity of 90.1% and specificity of 49.1%, whereas the role functioning TCI (≤ 37) showed sensitivity of 60.4% and specificity of 88.2%. Among symptom domains, fatigue (≥ 57) showed sensitivity of 79.1% and specificity of 67.8%, whereas sleep disturbances (≥ 55) showed sensitivity of 77.3% and specificity of 36.0%. Because the published TCIs were derived using domain-specific relevance anchors, these differences were interpreted as reflecting anchor dependence rather than superiority of either threshold set.

Form-stratified sensitivity analyses for the five domains with different short and long forms showed similar discriminatory performance to the pooled analyses, with AUC differences of -0.02 to 0.03. Exact Youden-optimal cut-points varied by form in some domains, particularly cognitive functioning (long ≤ 38.8 vs. short ≤ 43.9, pooled ≤ 41.5) and pain (long ≥ 58.3 vs. short ≥ 57.6, pooled ≥ 55.9) (Table S8).

## Discussion

This post hoc analysis estimated EQ-VAS-associated cut-points for EORTC CAT Core short-form scores and compared them descriptively with published relevance-based TCIs. Discrimination varied substantially across domains (AUC 0.57–0.82): eight domains had AUC ≥ 0.70, whereas diarrhea showed low discrimination. Four domains did not meet the criterion used for interpretation of |ρ| ≥0.30 and were therefore interpreted as exploratory.

EQ-VAS and CAT Core were completed by the same patients at the same baseline assessment. Consequently, these analyses quantify cross-sectional convergence between overlapping patient-reported constructs rather than validation against an independent criterion of clinical importance. EQ-VAS reflects global self-rated health, whereas patient-reported ECOG captures performance status. Moreover, patient-reported ECOG is not equivalent to routinely clinician-recorded ECOG [15, 20], and EQ-VAS is available only when EQ-5D is collected. These measures are therefore better considered pragmatic trial-based reference classifications than routine clinical relevance anchors.

Administration format also affects interpretation. PRO B used fixed CAT Core short forms rather than adaptive item selection, and different baseline forms were administered for several domains (Table S1). Several symptom domains also had few distinct observed T-scores and pronounced concentration at the minimum score (Table S3 and Fig. 1). In such settings, a cut-point may partly distinguish absence or minimal presence of a symptom from any symptom rather than finely grade severity, and the wide bootstrap intervals for some cut-points reflect this uncertainty. Form-stratified analyses showed similar discrimination across forms, although the exact optimal cut-point differed for some domains, particularly cognitive functioning and pain, suggesting some sensitivity of threshold location to the administered form despite calibration to a common T-score metric. Transferability of these cut-points to adaptive CAT administration or other fixed forms therefore requires further evaluation.

Where adequately supported, these cut-points may aid interpretation of individual patient scores at a single time point. They should not be used to dichotomize group-level HRQoL outcomes or replace analyses of continuous scores, and predictive values will depend on case mix. Given the domain-level variation in anchor association and discrimination, these values should be regarded as context-specific reference points rather than strict clinical cut-offs [8].

### Limitations

Several limitations should be considered when interpreting these findings. First, EQ-VAS ≤ 70 was a pragmatic global-health classification rather than a validated domain-specific clinical-importance threshold in mBC; similar findings using ≤ 65 do not establish clinical meaning. Second, EQ-VAS and CAT Core were patient-reported at the same assessment and therefore do not provide independent validation. Third, four domains showed weak anchor associations, and several symptom domains had pronounced floor concentrations and restricted score distributions, limiting cut-point stability. Fourth, fixed short forms rather than adaptive CAT administration were used, and different forms were administered for several domains; transferability to adaptive administration therefore requires evaluation. Fifth, this post hoc secondary analysis used complete-case data and requires external validation. Finally, the study population consisted of women with mBC treated in certified breast cancer centers in Germany, which may limit generalizability to other healthcare settings or cancer populations.

## Conclusions

This study derived EQ-VAS-associated cut-points for EORTC CAT Core short-form scores in metastatic breast cancer. These global-health-associated cut-points showed useful discrimination in some domains but weak support in others and should not be interpreted as replacements for published domain-specific relevance-based TCIs. Their use should be limited to context-specific individual-level interpretation when corresponding reference measures are available, with further validation required for adaptive CAT administration and other populations.

## Supplementary Information

Below is the link to the electronic supplementary material.

Supplementary Material 1

## Data Availability

No datasets were generated or analysed during the current study.

## Abbreviations

AUC: Area under the curve
CAT: Computer adaptive testing
ECOG: Eastern Cooperative Oncology Group
EORTC: European Organisation for Research and Treatment of Cancer
EQ-5D-5L: EuroQol 5-Dimension 5-Level questionnaire
EQ-VAS: EuroQol visual analogue scale
GH/QoL: Global health status/quality of life
HER2: Human epidermal growth factor receptor 2
HR: Hormone receptor
HRQoL: Health-related quality of life
mBC: Metastatic breast cancer
PRO: Patient-reported outcome
QLQ-C30: Quality of Life Questionnaire Core 30
ROC: Receiver operating characteristic
SD: Standard deviation
TCIs: Thresholds for clinical importance
TNBC: Triple-negative breast cancer
