# Supplementary material for: Interpreting EORTC CAT Core short-form scores in metastatic breast cancer: EQ-VAS-associated cut-points and comparison with published thresholds for clinical importance
Source: J Patient Rep Outcomes. 2026 Nov 6;10:207. doi: 10.1186/s41687-026-01254-3 (PMC13751971; doi:10.1186/s41687-026-01254-3)
Supplement: Supplementary file 1 [file 41687_2026_1254_MOESM1_ESM.docx]

**Supplementary**

**Interpreting EORTC CAT Core short-form scores in metastatic breast cancer: EQ-VAS-associated cut-points and comparison with published thresholds for clinical importance**

[**Table S1** Items included in the fixed EORTC CAT Core questionnaire versions used in the baseline analysis	2](#_Toc244591368)

[**Table S2** Baseline characteristics of participants with and without CAT Core data available for the baseline analysis	4](#_Toc244591369)

[**Table S3** Distribution of EORTC CAT Core T-scores used in the baseline analysis	5](#_Toc244591370)

[**Table S4** Bootstrap estimates and optimism-corrected performance of EQ-VAS-associated cut-points using EQ-VAS ≤70 as the primary reference classification	6](#_Toc244591371)

[**Table S5** Bootstrap estimates and optimism-corrected performance of ECOG-associated cut-points using ECOG ≥ 2 as the secondary reference classification	7](#_Toc244591372)

[**Table S6** Bootstrap estimates and optimism-corrected performance of EQ-VAS-associated cut-points using EQ-VAS ≤65 in sensitivity analysis	8](#_Toc244591373)

[**Table S7** Published EORTC CAT Core thresholds from Giesinger et al. applied to the PRO B cohort	9](#_Toc244591374)

[**Table S8** Form-stratified sensitivity analyses of EQ-VAS-associated cut-points using EQ-VAS ≤70	10](#_Toc244591375)

## **Table S1** Items included in the fixed EORTC CAT Core questionnaire versions used in the baseline analysis

| **EORTC CAT Core short forms** | **Short** | **Long** |
| --- | --- | --- |
| **Quality of Life (QL)** |  |  |
| QL1 | X | X |
| QL2 | X | X |
| **Physical Functioning (PF)** |  |  |
| PF15 | X | X |
| PF28 | **-** | X |
| PF24 | X | X |
| PF23 | **-** | X |
| PF19 | X | X |
| PF31 | **-** | X |
| PF13 | X | X |
| PF5 | X | X |
| **Role Functioning (RF)** |  |  |
| RF8 | X | X |
| RF4 | X | X |
| RF2 | X | X |
| RF9 | X | X |
| RF10 | X | X |
| RF5 | X | X |
| RF3 | X | X |
| RF7 | X | X |
| **Emotional Functioning (EF)** |  |  |
| EF1 | X | X |
| EF2 | **-** | X |
| EF8 | **-** | X |
| EF16 | X | X |
| EF10 | **-** | X |
| EF17 | X | X |
| EF19 | X | X |
| EF20 | **-** | X |
| **Cognitive Functioning (CF)** |  |  |
| CF11 | X | X |
| CF14 | **-** | X |
| CF34 | X | X |
| CF26 | **-** | X |
| CF13 | X | X |
| CF29 | **-** | X |
| CF18 | X | X |
| CF27 | **-** | X |
| **Social Functioning (SF)** |  |  |
| SF11 | X | X |
| SF1 | **-** | X |
| SF10 | X | X |
| SF8 | X | X |
| SF12 | **-** | X |
| SF3 | X | X |
| SF5 | **-** | X |
| SF9 | **-** | X |
| **Fatigue (FA)** |  |  |
| FA17 | X | X |
| FA24 | X | X |
| FA7 | X | X |
| FA25 | X | X |
| FA16 | X | X |
| FA22 | X | X |
| FA27 | X | X |
| FA31 | X | X |
| **Nausea and Vomiting (NV)** |  |  |
| NV1 | X | X |
| NV2 | X | X |
| **Pain (PA)** |  |  |
| PA16 | X | X |
| PA14 | X | X |
| PA2 | **-** | X |
| PA7 | **-** | X |
| PA9 | **-** | X |
| PA10 | **-** | X |
| PA8 | **-** | X |
| PA12 | **-** | X |
| **Dyspnea (DY)** |  |  |
| DY21 | X | X |
| DY25 | X | X |
| **Sleep quality (SL)** |  |  |
| SL5 | X | X |
| SL7 | X | X |
| **Appetite Loss (AP)** |  |  |
| AP4 | X | X |
| AP2 | X | X |
| **Constipation (CO)** |  |  |
| CO9 | X | X |
| CO2 | X | X |
| **Diarrhea (DI)** |  |  |
| DI12 | X | X |
| DI2 | X | X |
| **Financial Difficulties (FI)** |  |  |
| FI1 | X | X |
| FI6 | X | X |

X indicates that the item was included in the administered form; – indicates that the item was not administered. Fixed short forms derived from the EORTC CAT Core item banks were administered rather than computer-adaptive item selection. The forms administered in each version are shown above.

Short and long refer to the PRO B questionnaire versions used for the baseline analysis. Other intervention-group forms administered at weeks 2–4 were not included in the present analysis.

## **Table S2** Baseline characteristics of participants with and without CAT Core data available for the baseline analysis

| **Baseline characteristics** | **Total**  **(n = 924)** | **CAT Core data available**  **for baseline analysis** | | **p-value** |
| --- | --- | --- | --- | --- |
|  |  | **Yes (n = 855)** | **No (n = 69)** |  |
| Age at randomization (years) |  |  |  |  |
| Mean (SD) | 50.9 (11.0) | 51.0 (10.9) | 48.2 (12.8) | 0.065 |
| Median (min, max) | 50 (19, 83) | 50 (19, 83) | 49.5 (26, 73) |  |
| Missing | 15 | 0 | 15 |  |
| HR status |  |  |  | 0.22 |
| HR+/HER2− or HR+/HER2+ | 736 (79.7%) | 685 (80.1%) | 51 (73.9%) |  |
| HR-/HER2+ or HR-/HER2− | 188 (20.3%) | 170 (19.9%) | 18 (26.1%) |  |
| Site of metastases^1^ |  |  |  | 0.99 |
| Brain or multiple metastases | 515 (55.7%) | 477 (55.8%) | 38 (55.1%) |  |
| Bone, lymph nodes, or skin | 273 (29.5%) | 252 (29.5%) | 21 (30.4%) |  |
| Visceral (only one organ) | 136 (14.7%) | 126 (14.7%) | 10 (14.5%) |  |
| Metastatic presentation^2^ |  |  |  | 0.50 |
| De novo | 379 (41.0%) | 349 (40.8%) | 30 (43.5%) |  |
| Recurrent | 540 (58.4%) | 502 (58.7%) | 38 (55.1%) |  |
| Unknown | 5 (0.5%) | 4 (0.5%) | 1 (1.4%) |  |
| Endocrine therapy |  |  |  | 0.81 |
| No | 373 (41.0%) | 350 (40.9%) | 23 (42.6%) |  |
| Yes | 536 (59.0%) | 505 (59.1%) | 31 (57.4%) |  |
| Missing | 15 | 0 | 15 |  |
| Chemotherapy |  |  |  | 0.11 |
| No | 467 (51.4%) | 445 (52.0%) | 22 (40.7%) |  |
| Yes | 442 (48.6%) | 410 (48.0%) | 32 (59.3%) |  |
| Missing | 15 | 0 | 15 |  |
| Anti-HER2 therapy |  |  |  | 0.39 |
| No | 700 (77.0%) | 661 (77.3%) | 39 (72.2%) |  |
| Yes | 209 (23.0%) | 194 (22.7%) | 15 (27.8%) |  |
| Missing | 15 | 0 | 15 |  |
| Targeted therapy |  |  |  | 0.26 |
| No | 592 (65.1%) | 553 (64.7%) | 39 (72.2%) |  |
| Yes | 317 (34.9%) | 302 (35.3%) | 15 (27.8%) |  |
| Missing | 15 | 0 | 15 |  |
| Other therapy |  |  |  | 0.90 |
| No | 786 (86.5%) | 739 (86.4%) | 47 (87.0%) |  |
| Yes | 123 (13.5%) | 116 (13.6%) | 7 (13.0%) |  |
| Missing | 15 | 0 | 15 |  |

Percentages are based on participants with non-missing data for the respective characteristic; missing values are reported separately.

^1^ Site of metastases categories correspond to the metastatic-site stratification variable used in the PRO B trial.

^2^ Classification based on timing of first documented distant metastasis relative to initial diagnosis (≤90 days = de novo; >90 days = recurrent)

HER2, human epidermal growth factor receptor 2; HR, hormone receptor; SD, standard deviation

## **Table S3** Distribution of EORTC CAT Core T-scores used in the baseline analysis

| **Domain** | **Version** | **Attainable T-score** | | **Number of observations** | **Observed T-score** | | **Number at observed minimum** | **% at observed minimum** | **Number of unique scores** |
| --- | --- | --- | --- | --- | --- | --- | --- | --- | --- |
|  |  | **Minimum** | **Maximum** |  | **Minimum** | **Maximum** |  |  |  |
| Global health/QoL | Common | 22.3 | 68.2 | 855 | 22.4 | 68.2 | 9 | 1.1 | 13 |
| Physical functioning | Long | 14.3 | 63.2 | 464 | 19.8 | 63.2 | 2 | 0.4 | 162 |
|  | Short | 14.3 | 59.5 | 391 | 14.3 | 59.5 | 2 | 0.5 | 101 |
| Role functioning | Common | 16.5 | 58.7 | 855 | 16.5 | 58.7 | 15 | 1.8 | 219 |
| Emotional functioning | Long | 20.5 | 62.0 | 464 | 20.6 | 62.0 | 4 | 0.9 | 165 |
|  | Short | 24.0 | 61.5 | 391 | 24.0 | 61.5 | 13 | 3.3 | 37 |
| Cognitive functioning | Long | 16.2 | 58.4 | 464 | 16.4 | 58.4 | 4 | 0.9 | 162 |
|  | Short | 19.8 | 57.3 | 391 | 19.9 | 57.3 | 8 | 2.0 | 53 |
| Social functioning | Long | 17.0 | 57.5 | 464 | 20.0 | 57.5 | 1 | 0.2 | 176 |
|  | Short | 20.7 | 56.4 | 391 | 20.7 | 56.4 | 5 | 1.3 | 81 |
| Fatigue | Common | 36.8 | 78.3 | 855 | 36.8 | 78.3 | 30 | 3.5 | 210 |
| Nausea/vomiting | Common | 51.7 | 96.3 | 855 | 51.8 | 96.3 | 511 | 59.8* | 12 |
| Pain | Long | 39.6 | 77.7 | 464 | 39.6 | 77.6 | 74 | 15.9 | 142 |
|  | Short | 41.4 | 71.7 | 391 | 41.4 | 71.7 | 109 | 27.9 | 12 |
| Dyspnea | Common | 42.5 | 75.1 | 855 | 42.5 | 75.0 | 201 | 23.5 | 10 |
| Sleep disturbances | Common | 43.7 | 72.0 | 855 | 43.7 | 72.0 | 133 | 15.6 | 13 |
| Appetite loss | Common | 45.9 | 82.2 | 855 | 45.9 | 82.2 | 493 | 57.7* | 13 |
| Constipation | Common | 43.6 | 79.5 | 855 | 43.6 | 79.4 | 431 | 50.4* | 14 |
| Diarrhea | Common | 45.2 | 84.4 | 855 | 45.2 | 84.4 | 442 | 51.7* | 15 |
| Financial impact | Common | 46.1 | 80.9 | 855 | 46.1 | 80.9 | 510 | 59.6* | 15 |

* ≥50% of participants had the observed minimum score. For domains in which different fixed forms were administered at baseline by short or long versions, attainable and observed score distributions are presented separately by form. Domains with the same baseline form are presented as common version. The attainable range represents the minimum and maximum T-scores possible from all valid response patterns for the respective baseline form. Observed minimum and maximum represent scores occurring in the PRO B baseline sample.

## **Table S4** Bootstrap estimates and optimism-corrected performance of EQ-VAS-associated cut-points using EQ-VAS ≤70 as the primary reference classification

| **Domain** | **Cut-point**  **(95% bootstrap CI)** | **AUC**  **(95% bootstrap CI)** | **Sensitivity, % (95% bootstrap CI)** | **Corrected sensitivity, %** | **Specificity, % (95% bootstrap CI)** | **Corrected specificity, %** | **PPV, %**  **(95% bootstrap CI)** | **Corrected PPV, %** | **NPV, %**  **(95% bootstrap CI)** | **Corrected NPV, %** | **Youden J**  **(95% bootstrap CI)** | **Corrected Youden J** |
| --- | --- | --- | --- | --- | --- | --- | --- | --- | --- | --- | --- | --- |
| **Functioning domain** |  |  |  |  |  |  |  |  |  |  |  |  |
| Physical functioning | ≤41.9  (40.8, 44.4) | 0.82  (0.79, 0.84) | 77.9  (71.6, 85.0) | 77.5 | 73.0  (65.1, 79.0) | 72.6 | 76.4  (71.7, 80.8) | 76.0 | 74.7  (69.8, 80.5) | 74.3 | 0.510  (0.454, 0.569) | 0.501 |
| Role functioning | ≤38.1  (37.5, 41.3) | 0.82  (0.79, 0.85) | 66.7  (62.3, 83.4) | 65.9 | 84.6  (68.5, 89.1) | 83.8 | 82.9  (73.4, 87.3) | 82.1 | 69.4  (66.0, 80.0) | 68.7 | 0.513  (0.471, 0.578) | 0.497 |
| Emotional functioning | ≤39.9  (38.5, 43.5) | 0.74  (0.71, 0.78) | 64.9  (58.5, 81.9) | 64.1 | 73.6  (56.6, 80.0) | 72.7 | 73.3  (65.9, 78.0) | 72.5 | 65.2  (61.4, 75.6) | 64.3 | 0.384  (0.335, 0.452) | 0.367 |
| Cognitive functioning | ≤41.5  (38.2, 46.2) | 0.71  (0.67, 0.74) | 59.5  (43.1, 79.4) | 58.4 | 72.8  (53.5, 88.7) | 71.7 | 71.0  (63.8, 81.7) | 69.8 | 61.6  (56.9, 71.8) | 60.6 | 0.323  (0.280, 0.393) | 0.300 |
| Social functioning | ≤39.5  (37.8, 44.2) | 0.77  (0.74, 0.80) | 65.3  (55.7, 83.5) | 64.4 | 76.6  (58.4, 86.2) | 75.6 | 75.7  (68.5, 83.0) | 74.7 | 66.4  (61.7, 76.5) | 65.5 | 0.419  (0.374, 0.486) | 0.400 |
| **Symptom domain** |  |  |  |  |  |  |  |  |  |  |  |  |
| Fatigue | ≥59.0  (56.4, 59.8) | 0.80  (0.77, 0.83) | 71.8  (67.5, 83.8) | 71.3 | 77.8  (65.4, 82.2) | 77.2 | 78.4  (71.8, 82.4) | 77.8 | 71.2  (67.4, 79.2) | 70.6 | 0.497  (0.445, 0.559) | 0.484 |
| Nausea/vomiting | ≥54.8  (54.8, 61.1) | 0.65  (0.62, 0.69) | 53.8  (49.0, 58.4) | 53.7 | 75.1  (70.9, 79.7) | 75.0 | 70.7  (65.9, 75.8) | 70.6 | 59.2  (54.9, 63.5) | 59.2 | 0.289  (0.227, 0.353) | 0.287 |
| Pain | ≥55.9  (55.5, 58.9) | 0.72  (0.69, 0.76) | 62.2  (52.3, 74.4) | 61.6 | 75.6  (62.9, 85.6) | 74.9 | 74.0  (69.0, 82.0) | 73.3 | 64.1  (59.0, 69.5) | 63.6 | 0.377  (0.329, 0.445) | 0.365 |
| Dyspnea | ≥61.5  (53.8, 61.5) | 0.72  (0.69, 0.76) | 55.4  (52.5, 81.6) | 54.8 | 78.8  (52.7, 82.5) | 78.2 | 74.5  (63.7, 79.1) | 74.0 | 61.3  (57.9, 74.0) | 60.7 | 0.342  (0.296, 0.408) | 0.330 |
| Sleep disturbances | ≥59.2  (50.9, 65.3) | 0.61  (0.58, 0.65) | 47.5  (24.7, 82.3) | 46.6 | 68.5  (32.8, 89.9) | 67.4 | 62.8  (56.5, 74.3) | 61.5 | 53.9  (50.0, 64.0) | 52.9 | 0.160  (0.117, 0.231) | 0.140 |
| Appetite loss | ≥59.1  (52.3, 62.0) | 0.63  (0.60, 0.67) | 52.5  (38.1, 57.0) | 52.1 | 70.8  (67.6, 84.7) | 70.3 | 66.8  (63.0, 76.2) | 66.2 | 57.1  (52.1, 61.1) | 56.8 | 0.233  (0.178, 0.300) | 0.224 |
| Constipation | ≥54.2  (48.4, 66.1) | 0.60  (0.56, 0.63) | 48.2  (21.7, 59.3) | 47.5 | 67.5  (57.4, 92.0) | 66.7 | 62.4  (58.2, 77.6) | 61.3 | 53.8  (49.0, 58.7) | 53.2 | 0.157  (0.109, 0.226) | 0.142 |
| Diarrhea | ≥51.4  (51.4, 64.6) | 0.57  (0.54, 0.61) | 54.5  (27.5, 59.0) | 54.0 | 58.7  (54.9, 83.6) | 58.1 | 59.6  (55.8, 67.9) | 58.9 | 53.6  (48.6, 58.1) | 53.1 | 0.132  (0.075, 0.198) | 0.121 |
| Financial impact | ≥51.9  (51.9, 63.2) | 0.62  (0.59, 0.65) | 50.2  (27.7, 54.6) | 49.8 | 70.8  (67.3, 90.7) | 70.3 | 65.8  (61.6, 76.5) | 65.1 | 56.0  (51.2, 60.2) | 55.7 | 0.210  (0.153, 0.274) | 0.201 |

Values are based on EQ-VAS ≤70 defining lower global health status. Cut-points were selected by maximizing the Youden index.

For functioning domains, lower EORTC CAT Core scores indicate worse functioning and cut-points are expressed as ≤; for symptom domains, higher scores indicate greater symptom burden and cut-points are expressed as ≥.

95% percentile bootstrap confidence intervals were obtained using 5,000 bootstrap replications. AUC was calculated using the continuous CAT Core T-score and was not based on the dichotomized cut-point. For some domains with discrete score distributions, a bootstrap confidence limit coincided with the estimated cut-point. Optimism-corrected estimates were obtained by subtracting the mean bootstrap optimism from the apparent estimate. Bootstrap replications in which a unique Youden-optimal cut-point could not be identified because of ties were excluded; successful replications ranged from 4,988 to 4,999 of 5,000 across domains. PPV and NPV reflect the prevalence of EQ-VAS ≤70 in the study population and may differ in populations with different case mix.

AUC, area under the receiver operating characteristic curve; CAT, computer adaptive testing; CI, confidence interval; EQ-VAS, EuroQol visual analogue scale; EORTC, European Organisation for Research and Treatment of Cancer; NPV, negative predictive value; PPV, positive predictive value.

## **Table S5** Bootstrap estimates and optimism-corrected performance of ECOG-associated cut-points using ECOG ≥ 2 as the secondary reference classification

| **Domain** | **Cut-point**  **(95% bootstrap CI)** | **AUC**  **(95% bootstrap CI)** | **Sensitivity, % (95% bootstrap CI)** | **Corrected sensitivity, %** | **Specificity, % (95% bootstrap CI)** | **Corrected specificity, %** | **PPV, %**  **(95% bootstrap CI)** | **Corrected PPV, %** | **NPV, %**  **(95% bootstrap CI)** | **Corrected NPV, %** | **Youden J**  **(95% bootstrap CI)** | **Corrected Youden J** |
| --- | --- | --- | --- | --- | --- | --- | --- | --- | --- | --- | --- | --- |
| **Functioning domain** |  |  |  |  |  |  |  |  |  |  |  |  |
| Physical functioning | ≤41.5  (34.3, 42.0) | 0.80  (0.77, 0.84) | 86.3  (59.0, 91.2) | 84.5 | 59.8  (56.2, 87.4) | 59.3 | 40.4  (36.9, 60.3) | 39.4 | 93.3  (86.5, 95.6) | 92.6 | 0.461  (0.416, 0.534) | 0.438 |
| Role functioning | ≤36.5  (34.5, 38.2) | 0.80  (0.76, 0.83) | 73.2  (63.5, 83.3) | 72.0 | 74.7  (66.6, 83.7) | 74.4 | 47.8  (41.1, 56.0) | 47.0 | 89.8  (87.4, 93.2) | 89.4 | 0.479  (0.421, 0.552) | 0.464 |
| Emotional functioning | ≤38.4  (37.2, 43.7) | 0.71  (0.67, 0.75) | 64.9  (57.7, 84.3) | 63.4 | 68.3  (47.3, 74.7) | 67.8 | 39.2  (32.2, 45.5) | 38.4 | 86.0  (83.5, 90.9) | 85.4 | 0.331  (0.272, 0.413) | 0.312 |
| Cognitive functioning | ≤44.5  (37.7, 44.8) | 0.69  (0.64, 0.73) | 78.0  (49.0, 83.5) | 76.3 | 52.5  (49.0, 81.0) | 52.1 | 34.2  (30.8, 46.4) | 33.2 | 88.3  (82.5, 91.4) | 87.6 | 0.306  (0.250, 0.383) | 0.284 |
| Social functioning | ≤39.6  (36.4, 43.6) | 0.74  (0.70, 0.78) | 75.1  (57.5, 89.8) | 73.7 | 62.2  (45.6, 78.9) | 61.8 | 38.6  (33.2, 47.9) | 37.8 | 88.8  (84.8, 93.4) | 88.2 | 0.374  (0.316, 0.448) | 0.354 |
| **Symptom domain** |  |  |  |  |  |  |  |  |  |  |  |  |
| Fatigue | ≥61.5  (56.0, 63.4) | 0.76  (0.72, 0.79) | 69.3  (59.9, 90.1) | 67.8 | 70.0  (47.5, 78.6) | 69.5 | 42.1  (34.0, 50.0) | 41.3 | 87.8  (85.1, 94.0) | 87.2 | 0.392  (0.334, 0.466) | 0.373 |
| Nausea/vomiting | ≥67.0  (54.8, 67.0) | 0.65  (0.61, 0.70) | 39.5  (33.9, 64.2) | 38.6 | 87.1  (64.5, 89.5) | 86.8 | 49.1  (33.0, 56.9) | 48.4 | 82.0  (79.4, 86.1) | 81.7 | 0.266  (0.200, 0.342) | 0.255 |
| Pain | ≥58.1  (54.2, 63.5) | 0.71  (0.66, 0.75) | 63.4  (46.4, 83.8) | 61.7 | 68.7  (47.7, 84.4) | 68.2 | 39.0  (32.4, 52.3) | 37.8 | 85.6  (82.1, 90.7) | 85.0 | 0.321  (0.264, 0.407) | 0.298 |
| Dyspnea | ≥61.5  (61.5, 65.5) | 0.72  (0.68, 0.76) | 65.4  (52.5, 72.7) | 64.6 | 68.7  (65.1, 79.6) | 68.5 | 39.8  (34.7, 48.2) | 39.3 | 86.3  (82.9, 89.3) | 86.0 | 0.341 (0.276, 0.417) | 0.331 |
| Sleep disturbances | ≥57.7  (57.7, 61.5) | 0.62  (0.58, 0.66) | 62.9  (49.8, 69.4) | 62.5 | 59.8  (56.2, 71.0) | 59.6 | 33.1  (28.7, 38.4) | 32.8 | 83.6  (80.1, 86.9) | 83.5 | 0.227  (0.152, 0.303) | 0.221 |
| Appetite loss | ≥59.1  (52.3, 64.8) | 0.67  (0.63, 0.71) | 62.9  (38.2, 69.3) | 61.8 | 65.0  (62.0, 88.4) | 64.7 | 36.2  (32.5, 51.2) | 35.4 | 84.7  (81.0, 87.7) | 84.3 | 0.279  (0.217, 0.360) | 0.265 |
| Constipation | ≥54.2  (48.4, 54.2) | 0.63  (0.58, 0.67) | 59.0  (52.4, 69.9) | 58.7 | 64.6  (54.3, 68.4) | 64.4 | 34.5  (29.4, 39.6) | 34.2 | 83.3  (80.3, 86.7) | 83.2 | 0.236  (0.163, 0.312) | 0.231 |
| Diarrhea | ≥51.4  (51.4, 64.6) | 0.56  (0.52, 0.60) | 57.1  (25.8, 63.7) | 56.4 | 54.5  (51.1, 82.2) | 54.3 | 28.4  (24.6, 34.6) | 27.9 | 80.1  (76.2, 83.7) | 79.8 | 0.116  (0.046, 0.191) | 0.107 |
| Financial impact | ≥62.9  (51.9, 66.9) | 0.64  (0.59, 0.68) | 47.3  (30.1, 61.8) | 46.2 | 75.8  (63.7, 89.7) | 75.4 | 38.2  (31.5, 52.9) | 37.2 | 82.0  (78.8, 85.5) | 81.6 | 0.231  (0.166, 0.309) | 0.216 |

Values are based on ECOG ≥ 2 defining impaired health performance status. Cut-points were selected by maximizing the Youden index.

For functioning domains, lower EORTC CAT Core scores indicate worse functioning and cut-points are expressed as ≤; for symptom domains, higher scores indicate greater symptom burden and cut-points are expressed as ≥.

95% percentile bootstrap confidence intervals were obtained using 5,000 bootstrap replications. AUC was calculated using the continuous CAT Core T-score and was not based on the dichotomized cut-point. For some domains with discrete score distributions, a bootstrap confidence limit coincided with the estimated cut-point. Optimism-corrected estimates were obtained by subtracting the mean bootstrap optimism from the apparent estimate. Bootstrap replications in which a unique Youden-optimal cut-point could not be identified because of ties were excluded; successful replications ranged from 4,992 to 5,000 across domains. PPV and NPV reflect the prevalence of ECOG ≥ 2 in the study population and may differ in populations with different case mix.

AUC, area under the receiver operating characteristic curve; CAT, computer adaptive testing; CI, confidence interval; ECOG, Eastern Cooperative Oncology Group; EORTC, European Organisation for Research and Treatment of Cancer; NPV, negative predictive value; PPV, positive predictive value.

## **Table S6** Bootstrap estimates and optimism-corrected performance of EQ-VAS-associated cut-points using EQ-VAS ≤65 in sensitivity analysis

| **Domain** | **Cut-point**  **(95% bootstrap CI)** | **AUC**  **(95% bootstrap CI)** | **Sensitivity, % (95% bootstrap CI)** | **Corrected sensitivity, %** | **Specificity, % (95% bootstrap CI)** | **Corrected specificity, %** | **PPV, %**  **(95% bootstrap CI)** | **Corrected PPV, %** | **NPV, %**  **(95% bootstrap CI)** | **Corrected NPV, %** | **Youden J**  **(95% bootstrap CI)** | **Corrected Youden J** |
| --- | --- | --- | --- | --- | --- | --- | --- | --- | --- | --- | --- | --- |
| **Functioning domain** |  |  |  |  |  |  |  |  |  |  |  |  |
| Physical functioning | ≤41.9  (38.1, 42.0) | 0.81  (0.78, 0.84) | 81.3  (64.5, 84.8) | 80.3 | 67.6  (64.7, 84.3) | 66.9 | 66.2  (63.2, 77.1) | 65.4 | 82.2  (74.1, 85.6) | 81.4 | 0.489  (0.442, 0.554) | 0.472 |
| Role functioning | ≤38.1  (36.5, 41.2) | 0.82  (0.79, 0.85) | 71.3  (64.4, 85.5) | 70.5 | 80.1  (65.4, 86.5) | 79.5 | 73.7  (64.5, 79.7) | 72.9 | 78.1  (74.3, 85.7) | 77.5 | 0.514  (0.464, 0.575) | 0.500 |
| Emotional functioning | ≤39.9  (37.4, 43.5) | 0.75  (0.72, 0.78) | 68.6  (55.5, 83.0) | 67.5 | 70.3  (55.5, 83.0) | 69.6 | 64.4  (58.2, 73.3) | 63.4 | 74.1  (68.8, 81.1) | 73.3 | 0.389  (0.338, 0.457) | 0.371 |
| Cognitive functioning | ≤41.3  (38.3, 46.2) | 0.71  (0.67, 0.74) | 62.1  (45.2, 80.6) | 60.9 | 69.9  (51.5, 86.2) | 69.0 | 61.7  (55.0, 73.2) | 60.5 | 70.2  (65.5, 78.5) | 69.3 | 0.320  (0.274, 0.390) | 0.299 |
| Social functioning | ≤39.5  (38.0, 40.9) | 0.77  (0.73, 0.80) | 69.9  (62.3, 75.9) | 69.4 | 73.5  (67.9, 80.3) | 73.1 | 67.4  (62.4, 72.9) | 66.9 | 75.8  (71.7, 79.9) | 75.4 | 0.434  (0.375, 0.496) | 0.426 |
| **Symptom domain** |  |  |  |  |  |  |  |  |  |  |  |  |
| Fatigue | ≥59.0  (56.4, 59.8) | 0.79  (0.76, 0.82) | 75.1  (70.4, 84.9) | 74.5 | 72.5  (61.8, 77.3) | 72.0 | 68.1  (62.5, 73.1) | 67.6 | 78.8  (75.1, 84.5) | 78.3 | 0.475  (0.422, 0.540) | 0.465 |
| Nausea/vomiting | ≥54.8  (54.8, 61.1) | 0.65  (0.62, 0.69) | 56.1  (50.8, 61.2) | 56.0 | 72.2  (68.1, 76.5) | 72.2 | 61.2  (55.9, 66.7) | 61.1 | 67.8  (63.6, 71.8) | 67.7 | 0.283  (0.216, 0.349) | 0.282 |
| Pain | ≥55.9  (55.8, 63.5) | 0.73  (0.70, 0.77) | 66.7  (48.1, 71.2) | 66.0 | 73.1  (70.0, 89.7) | 72.7 | 66.0  (61.9, 78.6) | 65.3 | 73.7  (68.3, 77.5) | 73.3 | 0.398  (0.340, 0.462) | 0.387 |
| Dyspnea | ≥61.5  (56.6, 61.5) | 0.71  (0.68, 0.75) | 58.0  (52.8, 83.2) | 57.4 | 75.4  (49.8, 80.3) | 75.0 | 64.8  (54.4, 70.3) | 64.3 | 69.7  (66.3, 80.5) | 69.2 | 0.334  (0.282, 0.400) | 0.323 |
| Sleep disturbances | ≥57.7  (56.1, 65.3) | 0.63  (0.59, 0.67) | 56.6  (29.9, 62.2) | 55.9 | 63.1  (59.4, 87.8) | 62.5 | 54.6  (50.6, 64.9) | 53.7 | 65.1  (60.3, 69.6) | 64.5 | 0.198  (0.146, 0.269) | 0.184 |
| Appetite loss | ≥59.1  (59.1, 62.0) | 0.64  (0.61, 0.68) | 55.6  (41.1, 60.2) | 55.1 | 69.5  (66.6, 83.4) | 69.1 | 58.7  (55.0, 69.0) | 58.1 | 66.7  (61.8, 70.4) | 66.3 | 0.250  (0.199, 0.318) | 0.242 |
| Constipation | ≥60.3  (48.4, 60.3) | 0.59  (0.56, 0.63) | 27.6  (24.1, 61.1) | 26.7 | 87.3  (55.8, 90.1) | 86.6 | 63.0  (48.9, 69.8) | 61.8 | 60.7  (57.8, 67.0) | 60.0 | 0.149  (0.107, 0.219) | 0.133 |
| Diarrhea | ≥51.4  (51.4, 64.6) | 0.57  (0.54, 0.61) | 55.0  (25.9, 59.7) | 54.2 | 57.0  (54.3, 85.2) | 56.3 | 50.0  (46.9, 61.3) | 48.9 | 61.8  (57.0, 66.1) | 61.3 | 0.120  (0.073, 0.191) | 0.105 |
| Financial impact | ≥51.9  (51.9, 63.2) | 0.63  (0.60, 0.67) | 53.1  (31.1, 58.0) | 52.6 | 69.7  (66.5, 89.3) | 69.3 | 57.8  (53.5, 68.7) | 57.1 | 65.5  (61.1, 69.6) | 65.2 | 0.228  (0.171, 0.295) | 0.219 |

Values are based on EQ-VAS ≤65 defining lower global health status. Cut-points were selected by maximizing the Youden index.

For functioning domains, lower EORTC CAT Core scores indicate worse functioning and cut-points are expressed as ≤; for symptom domains, higher scores indicate greater symptom burden and cut-points are expressed as ≥.

95% percentile bootstrap confidence intervals were obtained using 5,000 bootstrap replications. AUC was calculated using the continuous CAT Core T-score and was not based on the dichotomized cut-point. For some domains with discrete score distributions, a bootstrap confidence limit coincided with the estimated cut-point. Optimism-corrected estimates were obtained by subtracting the mean bootstrap optimism from the apparent estimate. Bootstrap replications in which a unique Youden-optimal cut-point could not be identified because of ties were excluded; successful replications ranged from 4,994 to 5,000 across domains. PPV and NPV reflect the prevalence of EQ-VAS ≤65 in the study population and may differ in populations with different case mix.

AUC, area under the receiver operating characteristic curve; CAT, computer adaptive testing; CI, confidence interval; EQ-VAS, EuroQol visual analogue scale; EORTC, European Organisation for Research and Treatment of Cancer; NPV, negative predictive value; PPV, positive predictive value.

## **Table S7** Published EORTC CAT Core thresholds from Giesinger et al. applied to the PRO B cohort

| **Domain** | **Published Giesinger TCI** | **Sensitivity, %**  **(95% CI)** | **Specificity, %**  **(95% CI)** | **PPV, %**  **(95% CI)** | **NPV, %**  **(95% CI)** |
| --- | --- | --- | --- | --- | --- |
|  |  |  |  |  |  |
| **Functioning domain** |  |  |  |  |  |
| Physical functioning | ≤46 | 90.1 (87.0, 92.5) | 49.1 (44.2, 54.0) | 66.4 (62.6, 70.1) | 81.6 (76.2, 86.0) |
| Role functioning | ≤37 | 60.4 (55.7, 64.8) | 88.2 (84.6, 91.0) | 85.1 (80.7, 88.6) | 66.5 (62.4, 70.4) |
| Emotional functioning | ≤46 | 86.0 (82.5, 89.0) | 45.1 (40.3, 50.0) | 63.7 (59.7, 67.4) | 74.3 (68.4, 79.4) |
| Cognitive functioning | ≤45 | 71.2 (66.8, 75.2) | 58.9 (54.0, 63.7) | 66.0 (61.6, 70.1) | 64.6 (59.6, 69.4) |
| Social functioning | ≤41 | 69.8 (65.4, 73.9) | 70.8 (66.1, 75.0) | 72.8 (68.4, 76.8) | 67.7 (63.1, 72.0) |
| **Symptom domain** |  |  |  |  |  |
| Fatigue | ≥57 | 79.1 (75.0, 82.6) | 67.8 (63.0, 72.2) | 73.3 (69.1, 77.0) | 74.3 (69.6, 78.5) |
| Nausea/vomiting | ≥58 | 53.2 (48.5, 57.7) | 75.6 (71.1, 79.5) | 70.9 (65.8, 75.5) | 59.1 (54.7, 63.2) |
| Pain | ≥56 | 61.3 (56.7, 65.7) | 76.3 (71.9, 80.2) | 74.3 (69.6, 78.5) | 63.8 (59.4, 68.0) |
| Dyspnea | ≥60 | 55.4 (50.8, 60.0) | 78.8 (74.6, 82.6) | 74.5 (69.6, 78.9) | 61.3 (57.0, 65.4) |
| Sleep disturbances | ≥55 | 77.3 (73.1, 80.9) | 36.0 (31.5, 40.9) | 57.5 (53.5, 61.4) | 58.6 (52.3, 64.6) |
| Appetite loss | ≥63 | 41.2 (36.7, 45.9) | 81.4 (77.2, 84.9) | 71.2 (65.4, 76.4) | 55.3 (51.3, 59.3) |
| Constipation | ≥57 | 44.6 (40.0, 49.2) | 69.8 (65.1, 74.1) | 62.3 (56.8, 67.4) | 53.0 (48.7, 57.2) |
| Diarrhea | ≥62 | 35.1 (30.8, 39.7) | 76.3 (71.9, 80.2) | 62.4 (56.3, 68.2) | 51.3 (47.2, 55.3) |
| Financial impact | ≥58 | 48.9 (44.3, 53.5) | 71.5 (66.9, 75.8) | 65.8 (60.5, 70.7) | 55.6 (51.2, 59.8) |

Published thresholds for clinical importance were taken from Giesinger et al. and applied without modification to baseline EORTC CAT Core scores in the PRO B cohort. Their performance was evaluated against the EQ-VAS ≤70 reference classification, with EQ-VAS ≤70 defining lower global health status and >70 defining higher global health status. Thus, the sensitivity, specificity, PPV, and NPV reported here were calculated in PRO B and are not performance estimates reported in the original Giesinger study.

95% confidence intervals for sensitivity, specificity, PPV, and NPV were calculated using the Wilson method.

PPV and NPV depend on the prevalence of EQ-VAS ≤70 in the study population and may differ in populations with different case mix.

CAT, computer adaptive testing; CI, confidence interval; EQ-VAS, EuroQol visual analogue scale; EORTC, European Organisation for Research and Treatment of Cancer; NPV, negative predictive value; PPV, positive predictive value; TCI, threshold for clinical importance.

## **Table S8** Form-stratified sensitivity analyses of EQ-VAS-associated cut-points using EQ-VAS ≤70

| **Domain** | **Form** | **N analysis** | **Spearman ρ**  **(95% bootstrap CI) ^1^** | **Cut-point**  **(95% bootstrap CI)** | **AUC**  **(95% bootstrap CI)** | **Corrected sensitivity, %** | **Corrected specificity, %** |
| --- | --- | --- | --- | --- | --- | --- | --- |
| Physical functioning | Long | 453 | 0.566  (0.492, 0.631) | ≤41.8  (38.2, 44.5) | 0.81  (0.77, 0.85) | 74.0 | 71.9 |
|  | Short | 388 | 0.640  (0.572, 0.700) | ≤42.0  (42.0, 43.7) | 0.82  (0.78, 0.86) | 79.8 | 74.5 |
| Emotional functioning | Long | 453 | 0.498  (0.422, 0.568) | ≤41.5  (37.2, 43.5) | 0.76  (0.71, 0.80) | 80.8 | 59.2 |
|  | Short | 388 | 0.485  (0.401, 0.562) | ≤39.8  (38.8, 47.8) | 0.73  (0.68, 0.78) | 56.2 | 78.0 |
| Cognitive functioning | Long | 453 | 0.384  (0.304, 0.460) | ≤38.8  (37.7, 46.0) | 0.69  (0.64, 0.74) | 48.5 | 79.4 |
|  | Short | 388 | 0.457  (0.372, 0.536) | ≤43.9  (40.0, 49.3) | 0.74  (0.68, 0.78) | 64.4 | 70.8 |
| Social functioning | Long | 453 | 0.481  (0.400, 0.555) | ≤38.0  (37.4, 44.4) | 0.75  (0.71, 0.80) | 59.0 | 77.4 |
|  | Short | 388 | 0.565  (0.490, 0.631) | ≤39.5  (38.6, 42.9) | 0.79  (0.74, 0.83) | 62.3 | 82.4 |
| Pain | Long | 453 | -0.405  (-0.482, -0.324) | ≥58.3  (55.4, 63.5) | 0.70  (0.65, 0.75) | 56.2 | 77.8 |
|  | Short | 388 | -0.549  (-0.618, -0.475) | ≥57.6  (49.8, 61.9) | 0.75  (0.71, 0.80) | 53.2 | 84.0 |

Values are based on EQ-VAS ≤70 defining lower global health status. Cut-points were selected by maximizing the Youden index.

¹Spearman rank correlations were performed using the original continuous EQ-VAS scales.

The long and short forms were available for 464 and 391 participants, respectively; EQ-VAS-complete analyses included 453 and 388 participants. N analysis indicates the number with both CAT Core and EQ-VAS data.

For functioning domains, lower EORTC CAT Core scores indicate worse functioning and cut-points are expressed as ≤; for symptom domains, higher scores indicate greater symptom burden and cut-points are expressed as ≥.

95% percentile bootstrap confidence intervals were obtained using 5,000 bootstrap replications. AUC was calculated using the continuous CAT Core T-score and was not based on the dichotomized cut-point. For some domains with discrete score distributions, a bootstrap confidence limit coincided with the estimated cut-point. Optimism-corrected estimates were obtained by subtracting the mean bootstrap optimism from the apparent estimate.

AUC, area under the receiver operating characteristic curve; CAT, computer adaptive testing; CI, confidence interval; EQ-VAS, EuroQol visual analogue scale; EORTC, European Organisation for Research and Treatment of Cancer.
